# GTS-21, an α7-nicotinic acetylcholine receptor agonist, modulates Th1 differentiation in CD4^+^ T cells from patients with rheumatoid arthritis

**DOI:** 10.3892/etm.2014.1754

**Published:** 2014-06-03

**Authors:** SHIYAO WU, HONGJUN ZHAO, HUI LUO, XIANZHONG XIAO, HUALI ZHANG, TONG LI, XIAOXIA ZUO

**Affiliations:** 1 Department of Rheumatology, Xiangya Hospital, Central South University, Changsha, Hunan 410008, P.R. China; 2 Department of Pathophysiology, Xiangya School of Medicine, Central South University, Changsha, Hunan 410008, P.R. China

**Keywords:** GTS-21, Th1 cells, TBX21, α7nAchR, rheumatoid arthritis

## Abstract

GTS-21 (also known as DMBX-anabaseine), a selective α7 nicotinic acetylcholine receptor (α7nAChR) agonist, has previously been found to inhibit the inflammation associated with rheumatoid arthritis (RA). RA is an autoimmune disease, where an abnormal immune system plays a critical role in the occurrence and development of synovium inflammation and bone damage. However, prior to this study, the immunological mechanism by which GTS-21 protects against RA had not been elucidated. In the present study, the effects of GTS-21 on T helper 1 (Th1) cells, which have an important role in the inflammation associated with RA, were investigated. Peripheral blood mononuclear cells (PBMCs) and cluster of differentiation (CD)4^+^ T cells were separated from patients with RA, and the effects of GTS-21 on PBMCs stimulated with anti-CD3/-CD28 antibodies and CD4^+^ T cells were investigated in the context of Th1-cell differentiation. ELISA was used to analyze interferon (IFN)-γ expression and flow cytometric analysis was used to detect the percentage of IFN-γ^+^ CD3^+^CD8^−^ T cells. In addition, western blotting was employed to detect the levels of the T-box transcription factor TBX21, which is a Th1 cell-specific transcription factor. The present study showed that GTS-21 reduced IFN-γ production in PBMCs from patients with RA. Under conditions of Th1-cell differentiation, GTS-21 reduced the percentage of IFNγ^+^CD3^+^CD8^−^ T cells and IFN-γ production in the culture supernatant and also inhibited the expression of the Th1 cell-specific transcription factor TBX21. The effects of GTS-21 were blocked by the α7nAchR antagonist α-bungarotoxin, which increased the expression of IFN-γ and TBX21. This study demonstrated that GTS-21 is able to inhibit RA Th1-cell differentiation through activation of the α7nAchR.

## Introduction

Rheumatoid arthritis (RA) is a chronic inflammatory autoimmune disease, characterized by joint synovial inflammation and destruction of cartilage and bone. In the abnormal immune response associated with RA, the role of T cells, particularly cluster of differentiation (CD)4^+^ T cells, has always been of interest (1). T helper 1 (Th1) cells, as an important subtype of CD4^+^ T cells, have been previously recognized in the pathogenesis of RA. Previous studies have also shown that Th1 cells and the cytokine interferon (IFN)-γ play an important role in promoting inflammation in RA (2,3) and that inhibiting Th1 responses were testified to be valid in the patients with RA (4). IFN-γ is the hallmark cytokine of Th1 cells (5). In humans, Th1 cells can be differentiated from CD4^+^ T cells by activation of the Th1 cell-specific T-box transcription factor (TBX21) and IFN-γ genes under the stimulation of interleukin (IL)-12 and anti-IL-4 antibodies (6).

The vagus nerve can limit inflammation via the α7 nicotinic acetylcholine receptor (α7nAChR). GTS-21, also known as DMBX-anabaseine, is a selective α7nAChR agonist that has been demonstrated to inhibit serum tumor necrosis factor (TNF) and high-mobility group box 1 in a dose-dependent manner in mice with lethal endotoxemia and sepsis (7,8) and collagen-induced arthritis (9). *Ex vivo,* GTS-21 is able to reduce TNF production in RA whole blood cultures stimulated by endotoxin (10). To date, GTS-21 is one of the most well-characterized α7nAChR-specific agonists (11). Administration with GTS-21 in clinical trials was tolerated well by healthy volunteers and patients (12,13). GTS-21 exerts its effects by interacting directly with nAChRs. The α7nAChR, which is expressed in neurons and immune cells, has been proposed to have a role in anti-inflammatory and neuron-protective effects (9). Although GTS-21 has a protective effect in RA, the specific mechanism is not yet fully understood. It has been shown that α7nAChR is also located on the surface of T cells (14). To date, little attention has been focused on the effects of GTS-21 on Th1 cells in RA. Therefore, in the present study the effects of GTS-21 on Th1 cells from patients with RA were examined for the first time, to the best of our knowledge, and the preliminary molecular mechanism underlying the effects of GTS-21 was studied from the level of the α7nAchR.

## Materials and methods

### Patients

A total of 12 healthy volunteers and 10 patients who fulfilled the 2009 American College of Rheumatology revised criteria for RA (Tables I and II) were studied. A 28-joint Disease Activity Score (DAS28) was used to analyze the RA activity. (DAS28 = 0.56 × sqrt (number of tenderness joints) + 0.28 × sqrt (number of swollen joints) + 0.70 × Ln (ESR) + 0.014 × VAS (visual analogue scale), DAS28<2.6 remission; DAS28 2.6–3.2 low disease activity; DAS28 3.2–5.1 moderate disease activity; DAS28>5.1 high disease activity). ESR and CRP reflect the activity of RA. RF and anti-CCP antibodies are the relatively specific autoantibodies of RA. All of them are the items of the 2009 American College of Rheumatology revised criteria for RA. All of the subjects were treatment-naïve patients with RA and non-smokers. Patients who had complications were excluded. The study was approved by the Medical Ethics Committee of the Xiangya Hospital of Central South University (Changsha, China). All subjects signed the informed consent approved by the ethics committee.

### Cell preparation

Peripheral blood samples were obtained from patients with RA and healthy controls. The peripheral blood mononuclear cells (PBMCs) were separated from heparinized blood by density gradient centrifugation over Ficoll-Hypaque PLUS (GE Healthcare, Piscataway, NJ, USA). The CD4^+^ T cells were purified (>96%) from PBMCs using CD4^+^ T-cell Isolation Kit MicroBeads (Miltenyi Biotec, Bergisch-Gladbach, Germany), according to the manufacturer’s instructions.

### Cell culture and stimulation

Cells were suspended in RPMI-1640 medium supplemented with 10% fetal calf serum (FCS), 100 U/ml penicillin and 100 g/ml streptomycin at 37°C in a 5% CO_2_ atmosphere. The PBMCs (1×10^6^ cells/ml) were cultured for 72 h in 24-well plates and subsequently stimulated with anti-CD3 (3 μg/ml, clone HIT3a; BD Biosciences, Franklin Lakes, NJ, USA) and anti-CD28 (3 μg/ml, clone CD28.2; BD Biosciences) antibodies in the presence or absence of different concentrations (0.01, 0.1 or 1 μmol/l) of GTS-21 (Abcam, Cambridge, MA, USA). Purified CD4^+^ T cells (1×10^6^ cells/ml) were stimulated using anti-CD3-coated 96-well plates (BioCoat™ anti-human CD3 T-cell activation plates; BD Biosciences) plus anti-CD28 antibodies (3 μg/ml), in the presence of IL-12 (15 ng/ml, Peprotech, Inc., Rocky Hill, NJ, USA) and anti-IL-4 antibodies (4 μg/ml, Peprotech, Inc.) for Th1 differentiation for 72 h with GTS-21 (1 μmol/l) alone or combined with α-bungarotoxin (αBgt, 1 μmol/l; Sigma, St. Louis, MO, USA).

### Cell proliferation/viability analysis

The proliferation and viability of PBMCs and CD4^+^ T cells were analyzed with AlamarBlue^®^ assays (AbD Serotec, Kidlington, UK) (15,16). PBMCs from patients with RA were stimulated with anti-CD3/-CD28 antibodies and CD4^+^ T cells, which were cultured under Th1-promoting conditions, together with GTS-21 and/or αBgt at the indicated concentrations. After three days, cell proliferation/viability was monitored by AlamarBlue. A 100-μl/well sample of the cell suspension at a final concentration of 1×10^6^ cells/ml was seeded in triplicate in a 96-well microtiter plate, and 10 μl AlamarBlue was added to each well. The plates were then incubated at 37°C in a humidified 5% CO_2_ incubator for 8 h. AlamarBlue fluorescence was measured at an excitation wavelength of 544 nm and an emission wavelength of 590 mm (FLUOstar BMG Lab Technologies, Offenburg, Germany).

### Flow cytometric analysis

To enable the intracellular detection of cytokines, the Th1-differentiated CD4^+^ T cells were harvested and stimulated for 5 h with Leukocyte Activation Cocktail and BD GolgiPlug (containing PMA and ionomycinin; BD Biosciences). Cells were then stained extracellularly with phycoerythrin (PE) Cy5-conjugated CD3 monoclonal antibody (mAb) (eBiosciences, San Diego, CA, USA) and fluorescein isothiocyanate-conjugated CD8 mAb (eBiosciences), and subsequently fixed and permeabilized using a Cytofix/Cytoperm Fixation/Permeabilization kit (BD Biosciences). Intracellular staining with PE-conjugated IFN-γ mAb (eBiosciences) was then performed. Cell samples were acquired on a FACSCalibur flow cytometer and analyzed using CellQuest™ software (BD Biosciences).

### Western blot analysis

Purified CD4^+^ T cells (1×10^6^ cells/ml) were stimulated using anti-CD3-coated 96-well plates, in the presence of IL-12 (15 ng/ml) and anti-IL-4 antibody (4 μg/ml) for Th1 differentiation for 48 h with GTS-21 (1 μmol/l) alone or in combination with αBgt (1 μmol/l). Whole cell lysates were obtained from Th1-differentiated CD4^+^ T cells cultured in RPMI-1640 with 10% FCS, as described previously. Whole cell lysates were heated for 5 min at 90°C in 5× sodium dodecyl sulfate (SDS) loading buffer, fractionated by electrophoresis on SDS-polyacrylamide gels and transferred to polyvinylidene fluoride membranes. The membranes were blocked for 1 h at room temperature with 5% skimmed milk in 0.05% Tween 20/Tris buffered saline, followed by overnight incubation at 4°C with primary antibody (anti-TBX21 antibody, Abcam; anti-GAPDH antibody, Cell Signaling Technology, Inc., Danvers, MA USA). The blots were subsequently incubated with secondary goat anti-rabbit horseradish peroxidase-conjugated immunoglobulin G for 2 h at room temperature. The reaction was visualized by chemiluminescence detection. The bands of interest were quantified with Image-Pro Plus 6.0 (Media Cybernetics, Inc., Rockville, MD, USA) software.

### ELISA

The IFN-γ levels in the culture supernatants were measured using a human IFN-γ immunoassay quantikine ELISA (R&D Systems, Minneapolis, MN, USA). The cytokine concentration in the samples was calculated in pg/ml using recombinant human IFN-γ (R&D Systems) as a standard. Absorbance was measured at 450 nm with an ELISA plate reader, and the reading at 540 nm was subtracted to correct for optical imperfections in the plate.

### Statistics analysis

Data were analyzed with SPSS 17.0 statistical software, (SPSS, Inc., Chicago, IL, USA) and are expressed as the mean ± standard deviation. The significance of differences between the groups was determined with single-factor variance (one-way analysis of variance) followed by a multiple comparison test (Student-Newman-Keuls) unless stated otherwise. If the data did not satisfy the homogeneity of variance, the Kruskal Wallis test was used instead. The comparison between the cytokine production of patients with RA and that of healthy volunteers was analyzed using a nonparametric matched pairs test. P<0.05 was considered to indicate a statistically significant difference.

## Results

### Clinical data

Table I shows the demographic data of patients with RA and healthy volunteers. Patients and healthy volunteers were similar in age, although the percentage of females was higher among the patients with RA than that among the healthy volunteers. Table II shows the clinical and laboratory findings in treatment-naïve patients with RA. The patients with RA were suffering from a severely active form of the disease, as shown via the mean disease activity score in 28 joints value.

### Cell proliferation/viability analysis

**Figure 1:**
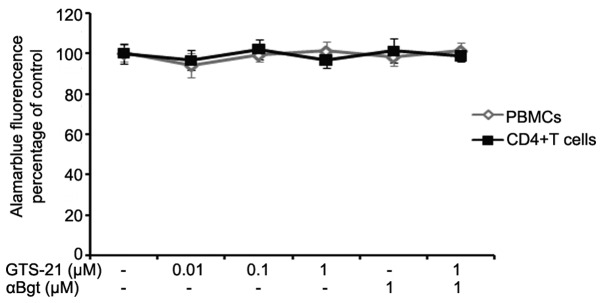
Cell proliferation/viability analysis. Each point is the mean ± standard deviation of three independent determinations. Comparison between PBMCs and CD4^+^ T cells, P>0.05. αBgt, α-bungarotoxin, PBMCs, peripheral blood mononuclear cells; CD4, cluster of differentiation 4.

PBMCs from patients with RA were stimulated with anti-CD3/-CD28 antibodies and CD4^+^ T cells, which were cultured under Th1-promoting conditions, together with GTS-21 and/or αBgt at the indicated concentrations. The effects of GTS-21, αBgt and the combination of the two on cell proliferation and viability were determined by AlamarBlue assays. Neither GTS-21, αBgt nor a combination of the two had any effect on the cell proliferation and viability of anti-CD3/-CD28-stimulated PBMCs or Th1-differentiated CD4^+^ T cells (P>0.05, Fig. 1).

### GTS-21 inhibits IFN-γ production by PBMCs from patients with RA

**Figure 2:**
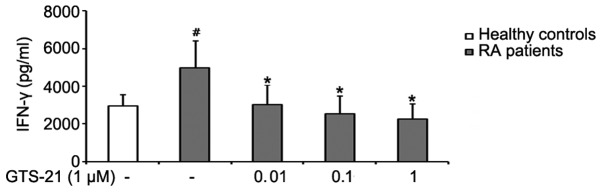
Effects of GTS-21 on IFN-γ production by PBMCs from patients with RA. Bars show the mean and standard deviation ELISA results from cultures of stimulated PBMCs from 10 patients with RA and 12 healthy controls. ^#^P<0.05 versus healthy controls; ^*^P<0.05 versus anti-CD3/-CD28-stimulated PBMCs from patients with RA without GTS-21 treatment. IFN, interferon; PBMCs, peripheral blood mononuclear cells; RA, rheumatoid arthritis, CD, cluster of differentiation.

PBMCs were cultured for 72 h with anti-CD3/-CD28 antibodies alone or with various concentrations of GTS-21. Anti-CD3/-CD28-stimulated PBMCs from patients with RA produced significantly more IFN-γ than stimulated PBMCs from healthy volunteers. GTS-21 inhibited the production of IFN-γ by PBMCs from patients with RA in a dose-dependent manner and reduced the levels of IFN-γ to levels similar to, or even below, those found in healthy volunteers (Fig. 2).

### GTS-21 reduces the percentages of IFN-γ^+^ T cells in RA CD4^+^ T cells during Th1 differentiation

**Figure 3:**
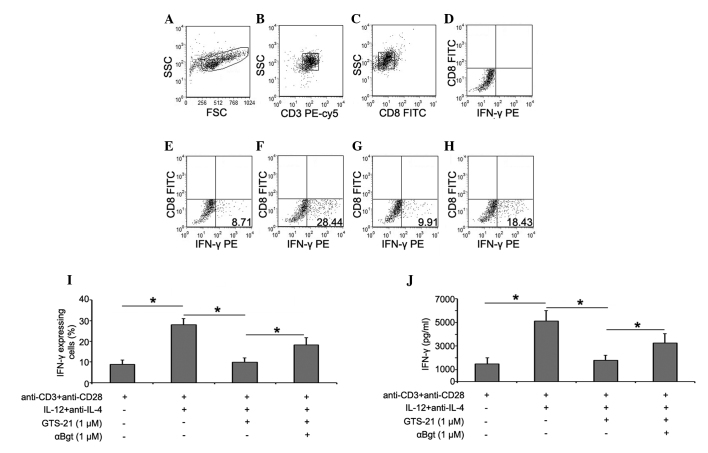
GTS-21 reduces the percentages of IFN-γ^+^ T cells in RA CD4^+^ T cells during Th1 differentiation. (A–C) Gating strategy used for fluorescence-activated cell sorting of CD3^+^CD8^−^ T cells from patients with RA. (D) Flow cytometric analysis isotype. (E) Percentages of IFN-γ^+^ cells among CD3^+^CD8^−^ T cells stimulated by anti-CD3/-CD28 for 72 h. (F) Percentages of IFN-γ^+^ cells among CD3^+^CD8^−^ T cells under Th1-differentiation conditions for 72 h. (G and H) Effect of (G) GTS-21 alone or (H) with αBgt on the expression of IFN-γ by CD3^+^CD8^−^ T cells from patients with RA. Representative results are shown. Numbers are the percentages of cells within the quadrants. (I) Quantification of the percentages of IFN-γ^+^CD3^+^CD8^−^ T cells. (J) Expression of IFN-γ in supernatants of differentiated Th1 CD4^+^ T cell cultures was analyzed by ELISA. Bars show the mean ± standard deviation. ^*^P<0.05. IFN, interferon; RA, rheumatoid arthritis; CD, cluster of differentiation ; αBgt, α-bungarotoxin; Th1, T helper 1; IL, interleukin; SSC, side scatter; FITC, fluorescein isothiocyanate; PE, phycoerythrin.

To examine the direct effects of GTS-21 on Th1 differentiation, CD4^+^ T cells were separated from PBMCs from patients with RA using CD4^+^ T-cell Isolation Kit MicroBeads and stimulated subsequently with GTS-21 under Th1-differentiation conditions. The intracellular expression of IFN-γ was detected by flow cytometry. Prior to flow cytometric analysis, it was necessary to augment the intracellular expression and block the extracellular secretion of IFN-γ using phorbol myristate acetate (PMA) and ionomycin. However, exposure to PMA leads to the internalization of membrane CD4 and to the loss of CD4^+^ T-cell resolution (17); therefore, the CD3^+^CD8^−^ T-cell population was used to represent the CD4^+^ T-cell population (18). The expression of IFN-γ in culture supernatant was detected by ELISA. The results showed that the percentage of IFN-γ^+^CD3^+^CD8^−^ T cells was increased significantly in the Th1-differentiation group. Incubation with GTS-21 (1 μmol/l) resulted in a reduction in the percentage of IFN-γ^+^CD3^+^CD8^−^ T cells. Following prestimulation with αBgt (1 μmol/l) prior to GTS-21 stimulation, IFN-γ expression during Th1 differentiation was increased (Fig. 3).

### GTS-21 reduces TBX21 levels in CD4^+^ T cells from patients with RA during Th1 differentiation

**Figure 4:**
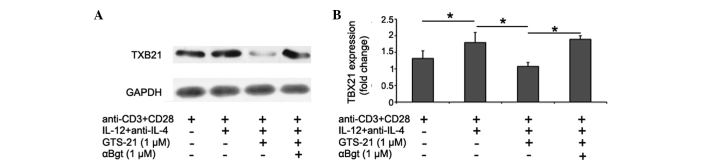
GTS-21 reduces TBX21 levels in CD4^+^ T cells from patients with RA during Th1 differentiation. (A) Representative images for the levels of TBX21 protein and GAPDH in each treatment group. (B) Statistical analysis results for the relative expression of TBX21 in each treatment group.^*^P<0.05. TBX21, T-box transcription factor TBX21; RA, rheumatoid arthritis; Th1, T helper 1; CD, cluster of differentiation; IL, interleukin; αBgt, α-bungarotoxin.

The effects of GTS-21 on the differentiation of Th1 cells from patients with RA were studied at the transcription factor level. The levels of the Th1 cell-specific transcription factor TBX21 in CD4^+^ T cells during Th1 differentiation were analyzed by western blotting. The data showed that TBX21 levels were increased during Th1 differentiation. GTS-21 (1 μmol/l) inhibited the expression significantly, and this effect was blocked by αBgt (1 μmol/l) (Fig. 4).

## Discussion

The present study aimed to investigate the effects of GTS-21 on the differentiation of Th1 cells using CD4^+^ T cells from patients with RA and to clarify the mechanism by which GTS-21 modulates the immune response, thus providing a novel basis for the treatment of RA with GTS-21.

In mammals, IFN-γ is known to be produced primarily by Th1 cells (5). Given that Th1 cells produce large quantities of IFN-γ, most Th1-mediated effects are attributed to this cytokine. RA is considered to be a Th1-driven autoimmune disease (2,3), and inhibiting Th1 differentiation could be a target in the treatment of RA to prevent arthritis and bone damage. In the present study, it was demonstrated that GTS-21 exerts beneficial effects on production of the Th1 cytokine IFN-γ by PBMCs from treatment-naïve patients with RA exhibiting high levels of disease activity. GTS-21 inhibits IFN-γ production by PBMCs at low concentrations (0.01, 0.1 and 1 μmol/l) and has its maximal IFN-γ-suppressive effect at a concentration of 1 μmol/l. AlamarBlue tests revealed that the inhibitory effect of GTS-21 on IFN-γ production by PBMCs is not mediated by inhibiting the proliferation/viability of cells. The results of this study are consistent with those of a previous study (19) and indicate that GTS-21 strongly downregulates IFN-γ production by PBMCs.

The present study demonstrated that GTS-21 has inhibitory effects on Th1 differentiation. Th1 cells are differentiated from CD4^+^ T cells (6). The Th1-cell differentiation system used in the present study included anti-CD3 and -CD28 antibodies, IL-12 and anti-IL-4 antibodies. In this study, it was found that GTS-21 inhibits Th1 differentiation by reducing the percentage of IFN-γ^+^CD3^+^CD8^−^ T cells and the levels of IFN-γ in Th1-differentiated CD4^+^ T-cell culture supernatant and also the expression of the Th1-specific transcription factor TBX21. AlamarBlue tests revealed that the inhibitory effect of GTS-21 on Th1-cell differentiation is not mediated by inhibiting the proliferation/viability of cells.

αBgt is an nAchR antagonist that binds to the α7 and α9nAchR, as well as the muscle-type nAchR; previous studies have demonstrated that T cells do not express the mRNA of α9nAchR and muscle-type nAchR (20,21). The present study showed that the effects of GTS-21 on Th1-cell differentiation can be counteracted by αBgt. This indicates that GTS-21 affects Th1 differentiation by means of its role in activating the α7nAchR.

Nicotine, a partial α7nAChR agonist, has also been found to exert anti-inflammatory effects in multiple diseases (22,23). Notably, nicotine also plays a protective role in experimental arthritis and RA (24). A previous study showed that nicotine can reduce the degree of joint inflammation in collagen-induced arthritic mice and that it is also capable of reducing the serum levels of IFN-γ and IL-6 secreted from RA fibroblast-like synoviocytes (25). GTS-21 is a selective α7nAChR agonist. Due to the obvious limitations of the therapeutic value of nicotine, as a result of its pharmacological nonspecificity and toxic side effects, selective α7nAChR agonists may be more favorable candidates for development as a novel medication for the treatment of RA.

This is the first study, to the best of our knowledge, to clarify the inhibitory effects of GTS-21 on Th1-cell differentiation by CD4^+^ T cells from patients with RA. From this novel perspective, the present study has elucidated the role of GTS-21 in the immune regulation of RA, thus providing a new basis for the future treatment of RA.

## Figures and Tables

**Table I:** Basic demographic data of patients with RA and controls.

Characteristic	Patients with RA	Controls
No. of subjects, n	10	12
Age, mean (SD)	42.3 (9.3)	39.1 (9.7)
Male/female, n/n	2/8	4/8

RA, rheumatoid arthritis; SD, standard deviation.

**Table II:** Clinical characteristics of patients with rheumatoid arthritis.

Characteristic	Result
DAS28, mean (SD)	6.51 (1.2)
Anti-CCP-positive, n (%)	7 (70)
RF-positive, n (%)	8 (80)
ESR in mm/h, mean (SD)	46.5 (33.7)
CRP in mg/l, mean (SD)	45.3 (31.1)

DAS28, disease activity score in 28 joints; CCP, cyclic citrullinated peptide; RF, rheumatoid factor; ESR, erythrocyte sedimentation rate; CRP, C-reactive protein, SD, standard deviation.
